# Catalytic Conversion of Levulinic Acid into 2-Methyltetrahydrofuran: A Review

**DOI:** 10.3390/molecules29010242

**Published:** 2024-01-02

**Authors:** Sreedhar Gundekari, Sanjib Kumar Karmee

**Affiliations:** 1 Department of Engineering Chemistry, Koneru Lakshmaiah Education Foundation, KL (Deemed to be) University, R.V.S Nagar, Moinabad-Chilkur Rd, Aziznagar 500075, Telangana, India; 2 The Odisha Renewable Energy Research Institute (ORERI), Subarnapur 767018, Odisha, India

**Keywords:** biomass, furanic molecules, levulinic acid, 2-methyltetrahydrofuran, hydrogenation

## Abstract

Biomass-derived furanics play a pivotal role in chemical industries, with 2-methyltetrahydrofuran (2-MTHF), a hydrogenated product of levulinic acid (LA), being particularly significant. 2-MTHF finds valuable applications in the fuel, polymer, and chemical sectors, serving as a key component in P-series biofuel and acknowledged as a renewable solvent for various chemical processes. Numerous research groups have explored catalytic systems to efficiently and selectively convert LA to 2-MTHF, using diverse metal-supported catalysts in different solvents under batch or continuous process conditions. This comprehensive review delves into the impact of metal-supported catalysts, encompassing co-metals and co-catalysts, on the synthesis of 2-MTHF from LA. The article also elucidates the influence of different reaction parameters, such as temperature, type and quantity of hydrogen source, and time. Furthermore, the review provides insights into reaction mechanisms for all documented catalytic systems.

## 1. Introduction

Furanic molecules, also known as furans, are a significant group of organic compounds distinguished by a cyclic structure consisting of a five-membered ring composed of four carbon atoms and one oxygen atom. These compounds are known to have diverse applications in the chemical industries, and their significance is heightened by their potential as renewable chemicals obtained from biomass [1,2]. Biomass materials, viz. wood, agricultural remnants, and plant substances, act as a sustainable reservoir for generating furanic molecules. The conversion of biomass into furans involves a spectrum of chemical processes, including dehydration and decarboxylation reactions [3,4]. The utilization of biomass as a precursor is advantageous due to its renewable nature, and in the context of diminishing reliance on fossil-derived fuels and chemicals [5,6]. Furanic molecules from biomass play a crucial role in the realms of green chemistry and the sustainable development of society. They serve as basic components for producing a variety of valuable substances and materials, spanning pharmaceuticals, polymers, resins, and solvents [7]. Moreover, furanic compounds possess the potential to supplant conventional petrochemical-based products, thereby fostering a more ecologically balanced and sustainable future [8]. In the pursuit of environmentally benign practices, researchers and scientists are continuously exploring novel techniques to effectively extract furanic molecules from bio-resources [9]. These endeavors aim to optimize conversion methods and discover new applications for these bio-based compounds. By unlocking the potential of furanic molecules from biomass, one can actively contribute to shaping the chemical industries that are not only sustainable but also environmentally vigilant [10]. Among the biomass-based furanic compounds, 2-hydroxymethylfurfural (HMF) and furfural play an important role in building a biorefinery. HMF and furfural are furanic compounds synthesized from biomass that have garnered significant attention among the scientific and industrial communities due to their diverse applications and potential as renewables [11,12].

HMF, a valuable furanic compound, is derived from sugars obtained from biomass, such as glucose, and fructose. Its production typically involves acid-catalyzed dehydration of the mentioned sugars [13,14]. Furfural, another valuable furanic compound derived from biomass, is primarily sourced from agricultural residues, such as corn cobs, oat hulls, and sawdust. Its production often involves the acid-catalyzed dehydration of pentose sugars, such as xylose [15]. Several factors, including the type and concentration of the acid catalyst, reaction temperature, and reaction time, can influence the efficiency of the reaction and the yield of HMF and furfural from sugars. It is crucial to optimize these reaction parameters to maximize the yield of HMF and furfural while minimizing the formation of other products [16,17]. Moreover, ongoing research explores novel catalytic processes and alternative feedstocks with the aim of improving the efficiency and sustainability of HMF and furfural production methods.

HMF and furfural serve as precursors for the synthesis of Levulinic acid (LA) [18]. LA plays a crucial role as a primary precursor in catalytic transformations for the production of fuels and chemicals [19,20]. LA, characterized by the molecular formula C_5_H_8_O_3_, can be obtained as a white crystalline solid below 30 °C. It exhibits solubility in both water-based solutions and typical organic solvents, including polar variants [19].

**Scheme 1:**
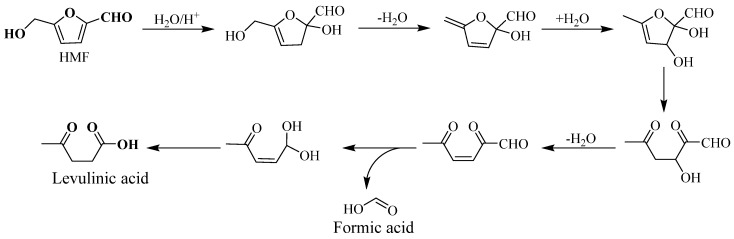
Mechanism of the reaction for synthesizing LA from HMF. Scheme taken from Gundekari et al. [20].

**Scheme 2:**
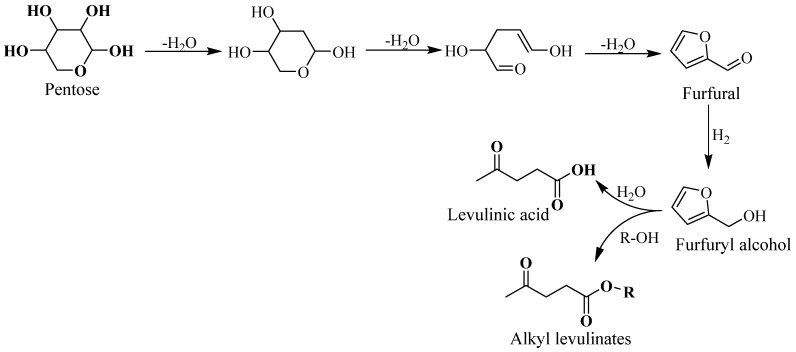
Mechanism of the reaction for synthesizing LA from furfural through the intermediate furfuryl alcohol. Scheme taken from Gundekari et al. [20].

LA is produced from glucose derived from cellulose in a two-step process. Initially, glucose undergoes isomerization to fructose, which is then dehydrated to generate HMF. In the subsequent step, HMF undergoes hydrolysis in an aqueous environment, resulting in the formation of LA with formic acid produced as a byproduct (Scheme 1) [21]. In the presence of an alcoholic medium, conversion leads to the formation of alkyl levulinates and alkyl formates [19]. LA can also be synthesized from furfural through an intermediate step involving furfuryl alcohol. Furfural is selectively hydrogenated to furfuryl alcohol using metal-supported catalysts. In the presence of water, furfuryl alcohol is transformed into LA and levulinic esters (alkyl levulinates) while using an alcoholic medium (Scheme 2) [22]. In general, the conversion of hydroxymethyl furfural (HMF) and furfuryl alcohol into LA is catalyzed by different acid catalysts, viz. solid acids, acidic ionic liquids, and mineral acids [23].

**Scheme 3:**
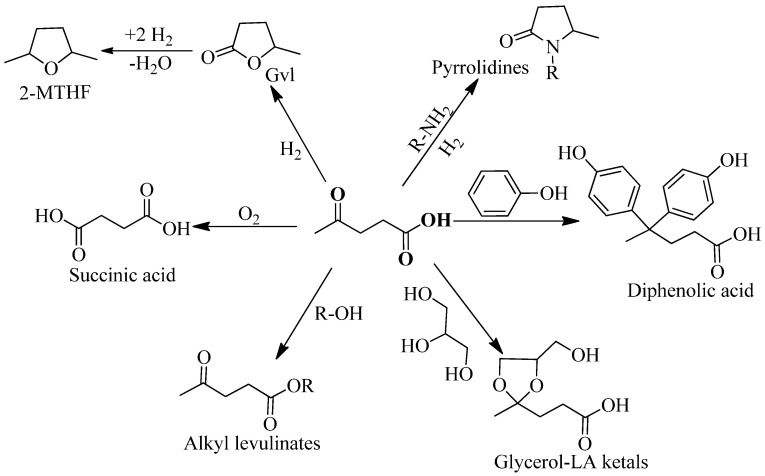
Catalytic organic transformations of LA. Scheme taken from Gundekari et al. [20].

LA possesses diverse functional groups, including carboxyl, carbonyl, and methylene, rendering it highly versatile and suitable for a wide range of organic reactions. These reactions include hydrocyclization, hydrogenation, dehydration, oxidation, reductive amination, halogenation, alkylation, and condensation, as illustrated in Scheme 3. LA can act as an intermediate in the synthesis of many compounds, including arylated cyclic lactones, alkylated lactones, ketals, halo substituted keto carboxylic acids, amides (cyclic/acyclic), valerates, valeric acids, aliphatic alcohols, acid halides, 1,4-diketones, alkanes, alkenes, cyclic ethers, and numerous other derivatives [24,25,26,27]. LA contains oxygen functionality (oxo and carboxylic), leading to the production of numerous high-value products through hydrocyclization and hydrodeoxygenation reactions [28]. When exposed to the H_2_ environment, LA primarily yields valuable products like 2-methyltetrahydrofuran (2-MTHF), 1,4-pentanediol (1,4-PDO), and γ-valerolactone (Gvl). The particular product is contingent on the catalytic system and the parameters of the reaction [29,30,31,32].

**Scheme 4:**
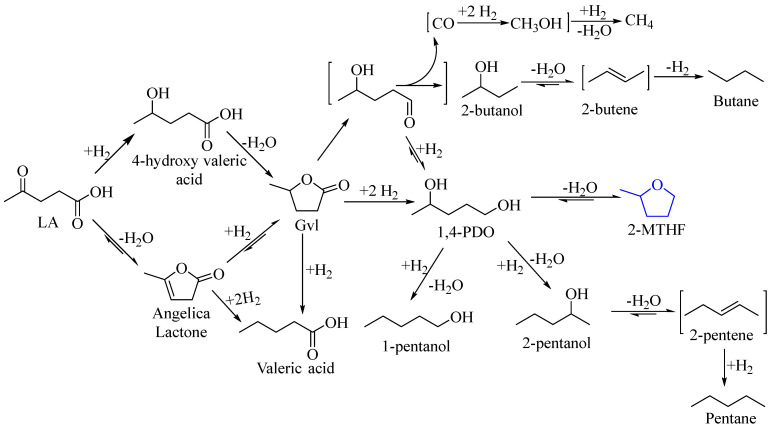
Reaction network of LA under H_2_ environment. Scheme adopted from [50].

The hydrogenated derivatives of LA play a crucial role in industry. For example, Gvl is a valuable platform chemical used in diverse applications, such as the preparation of biofuels like valerates, non-functionalized aromatic hydrocarbons, butanes, and liquid alkanes (Scheme 4) [33,34,35,36]. It also acts as an intermediate for polymers like 1,4-PDO, adipic acid, and α-methylene-γ-valerolactone [37,38,39]. Additionally, Gvl serves as an eco-friendly solvent in organic reactions, including the conversion of lignocellulose components [40,41]. Through sequential hydrogenation, LA transforms into 1,4-PDO via Gvl as an intermediate. Under high temperatures and acidic conditions, 1,4-PDO cyclizes to form 2-MTHF. 1,4-PDO also serves as a monomer in the production of essential polymers like polyesters, polyurethanes, and polyethers, which are utilized in textiles, adhesives, coatings, and flexible foams. Additionally, 1,4-PDO acts as a solvent in applications involving coatings, dyes, and printing inks [42]. Another hydrogenated product of LA, 2-MTHF, is gaining prominence in various industries due to its significant chemical properties [43,44,45].

This contribution focuses on catalytic methods for the conversion of LA under a hydrogen (H_2_) atmosphere, with an emphasis on the selective synthesis of 2-MTHF. The study thoroughly investigates and compiles the influence of diverse metal catalysts, catalytic supports, and reaction parameters pertaining to this conversion process, and documentation of the findings. The review concludes by highlighting insights of the subject by critically analyzing the reported catalytic systems, and challenges in this research area are also elaborated.

## 2. 2-Methyltetrahydrofuran (2-MTHF)

2-MTHF is a saturated cyclic ether, also known as a heterocyclic compound due to its oxygen atom within the ring structure. Chemically, it is represented as C_5_H_10_O, and its molecular formula indicates its close relation to tetrahydrofuran (THF), a commonly used solvent. As of 2021, the global market value of 2-MeTHF is anticipated to reach USD 3.4 billion, with a projected increase of USD 4.4 billion by 2030. Although its industrial production is currently on a modest scale, there is a growing demand for large-scale manufacturing. Envisaging an annual production of 9.1 million kg, a sizable production facility could potentially generate around USD 102 million in annual revenue. The US Department of Energy has classified 2-MTHF as a ‘P-Series fuel’ due to its impressive fuel characteristics. Its hydrophobic properties, high heating value, and elevated specific gravity allow it to be blended with gasoline up to 70% [46,47].

### 2.1. Preparation of 2-MTHF from LA

Typically, the manufacturing of 2-MTHF involves two separate methods: sequential hydrogenation of furfural and LA. In the first process, the furfural undergoes hydrogenation to produce 2-methylfuran (2-MF), which is subsequently further hydrogenated to result in 2-MTHF [48]. This reaction involves the introduction of hydrogen gas (H_2_) in the presence of suitable metals (such as Nickel, Palladium, Copper, Ruthenium) supported on appropriate materials like alumina (Al_2_O_3_), silica (SiO_2_), and activated carbon [49]. The production of 2-MTHF from LA comprises essential stages, such as hydrogenation, dehydration, and hydrocleavage processes, with key intermediates like Gvl and 1,4-PDO. Throughout this reaction, various side products might form, including valeric acid, pentanols, butanol, pentane, and butane (Scheme 4) [50]. In the initial stage, LA undergoes hydrocyclization, a process in which it cyclizes with the aid of a suitable catalyst. This stage leads to the generation of an intermediate compound, often Gvl. This intermediate, such as Gvl, then undergoes a hydrogenolysis reaction in which hydrogen gas (H_2_) is used to break the chemical bond (C-O). This process results in the formation of 1,4-PDO, which can undergo subsequent cyclization under acidic reaction conditions, ultimately yielding 2-MTHF as the desired end product (Scheme 4). The preparation of 2-MTHF from the two raw materials, LA and furfural, reveals that the conversion of LA to 2-MTHF is more efficient than furfural to 2-MTHF for several reasons. First, raw materials for LA, such as cellulose, are abundant compared to the xylose source used in furfural preparation. This abundance contributes to the effectiveness of the LA to 2-MTHF process. Additionally, the selectivity of 2-MTHF from LA is more pronounced compared to that from furfural. The hydrogenation of furfural to 2-MTHF generates various side products, including Cyclopentanone, furfuryl alcohol, tetrahydrofurfuryl alcohol, 2-methylfuran, tetrahydrofurfural, 1,2-pentanediol, and 1,5-pentanediol. On the other hand, the conversion of LA to 2-MTHF results in only Gvl and 1,4-pentanediol as side products, demonstrating a higher selectivity for 2-MTHF in this process. Furthermore, the conversion of furfural to 2-MTHF requires a substantial amount of H_2_ (4 equivalents), whereas the LA to 2-MTHF conversion necessitates only 3 equivalents of H_2_. This reduced hydrogen consumption adds to the overall efficiency of the LA to the 2-MTHF pathway.

The conversion of LA into 2-MTHF stands as a vibrant area of research within the fields of green chemistry and biofuels [51]. In recent research efforts, scientists have focused on investigating various catalysts, reaction conditions, and methods aimed at the efficient conversion of LA into 2-MTHF. Researchers have extensively investigated the use of metal catalysts, acid catalysts, and their combinations to facilitate this conversion. Furthermore, these investigations have involved a wide range of feedstocks, along with variations in reaction temperatures, pressures, and solvent systems, all aimed at optimizing the yield and selectivity of 2-MTHF. Section 3 provides a critical review of the conversion of LA to 2-MTHF using various catalysts.

In the hydroconversion of LA to 2-MTHF, batch experiments were carried out using a high-pressure reactor constructed from either Hastelloy C-276 or stainless steel (SS-316). The reactor underwent thorough examination to optimize the reaction parameters, including temperature, pressure, and reaction time. Initially, the reactor vessel was loaded with the catalyst. Following this, the substrate (LA) dissolved in either water or an organic solvent was introduced into the vessel. The system underwent three purges with nitrogen (N_2_) or hydrogen (H_2_) before pressurization/feeding with a fixed amount of H_2_. The reaction was then conducted at the desired temperature and duration. After completion, the reactor was cooled to room temperature, and excess H_2_ was released. The catalyst was separated through simple centrifugation. Quantitative analysis of the product mixture was typically carried out using Gas Chromatography (GC) and/or Gas Chromatography-Mass Spectrometry (GC-MS) [52].

In continuous hydrocyclization experiments converting LA to 2-MTHF, a stainless steel fixed-bed catalytic reactor was typically employed [53]. The continuous reactor setup included a pre-heater for vaporizing or heating the liquid feed, and an assembly comprising a reactor and a furnace in the upstream section. The downstream section comprised a condenser and a gas-liquid separator. The reactor tube featured temperature thermocouples with transmitters. The catalytic reactor bed was enclosed within a furnace and heated electrically. Initially, the reactor was loaded with the catalyst. The loaded catalyst was pre-heated at a specific temperature and duration until the physisorbed H_2_O molecules were removed from the catalyst. Various parameters such as column temperature, pre-heater temperature, liquid flow rate (for LA in solvent), hydrogen (H_2_) feed flow (monitored and controlled using a Mass Flow Controller), and H_2_ pressure in the reactor were systematically adjusted for optimizing the reaction. Under the optimized conditions, the reaction was carried out for a specific time to assess the stability and activity of the catalyst. Throughout the reaction, the product mixture was condensed and separated. The collected product was then analyzed using GC or GC-MS.

### 2.2. Properties of 2-MTHF

22-Methyltetrahydrofuran (2-MeTHF) is a chemical compound that possesses several important properties [54,55,56]. The key and important 2-MTHF properties are mentioned below. These properties make 2-MTHF suitable for a range of applications, including as a solvent in various chemical reactions [57], as a component in fuel formulations [58,59], and in other industrial processes.

2-MTHF is classified as a polar aprotic solvent, signifying its partial polarity due to the presence of an oxygen atom in its structure while lacking acidic hydrogen atoms. This characteristic renders it suitable for a broad spectrum of chemical reactions. Under typical conditions, 2-MTHF exhibits stability; however, its reactivity depends on the presence of specific reagents or catalysts in particular reaction setups [55]. Although generally considered to have low toxicity, prudent handling is essential, as with any chemical substance. It is notably less toxic than common solvents like tetrahydrofuran (THF) and dichloromethane (DCM). Regarded as environmentally friendly, 2-MTHF boasts a lower environmental impact than some counterparts and finds applications in green chemistry initiatives. Despite these advantages, it is crucial to handle 2-MTHF with care due to its flammability; it possesses a relatively low flash point, making it susceptible to ignition at moderate temperatures [60,61].

### 2.3. Applications of 2-MTHF

2-MeTHF plays a crucial role in the fuel industry, serving as a component in alternative fuels and a solvent for fuel-related processes. Specifically, it is utilized in P-series biofuels, a family of alternative fuels designed by the U.S. Department of Energy, to reduce greenhouse gases and promote renewable energy in transportation [62]. Additionally, 2-MeTHF functions as a solvent in the production of biofuels and can be used as a fuel additive to enhance combustion efficiency and reduce emissions [63,64]. It is also employed in fuel blending to improve properties like octane rating [64,65], and in fuel cell research, particularly in Li-ion batteries [66,67,68]. 2-MTHF serves as a green solvent in organic reactions, acting as an eco-friendly alternative to conventional solvents like tetrahydrofuran (THF) in various processes such as Grignard reactions and Suzuki-Miyaura cross-coupling reactions [55,57,69,70,71]. In the pharmaceutical industry, it finds application in peptide synthesis, aiding in solid-phase peptide synthesis and the preparation of peptide-based drugs [60,72]. Additionally, 2-MTHF is utilized for crystallization and purification of pharmaceutical compounds [63], dissolution testing in quality control [73,74], and as a solvent in the formulation of Active Pharmaceutical Ingredients (APIs) to enhance bioavailability [73,75,76]. In the agro-chemical industry, 2-MTHF is utilized as a solvent in the formulation of pesticides, herbicides, and insecticides, facilitating the dissolution and stabilization of active ingredients for effective and stable formulations [77]. Additionally, it finds application in the extraction and isolation of bioactive compounds from plant components, supporting the development of pesticides that use natural products as starting materials [54,78]. Moreover, in the field of electrochemistry, 2-MTHF serves as an electrolyte solvent, enhancing the performance and stability of electrochemical cells like lithium-ion batteries and fuel cells, particularly in improving low-temperature battery performance [67,68,79]. Additionally, it finds application as a solvent in various electrochemical techniques for redox reactions [80,81], electrodeposition, and electroplating processes [82]. 2-MTHF is also used in redox flow batteries, contributing to more efficient and cost-effective energy storage solutions [83], and as a reaction medium for electrochemical synthesis of organic compounds due to its favorable properties [84]. In the polymer industry, 2-MTHF serves as a versatile solvent for polymerization, contributing to the production of elastomers and thermoplastic polyurethanes [85]. It also functions as a polymerization initiator, controlling the properties of the formed polymers and enhancing the processability of certain thermoplastic polymers during extrusion and molding [86,87,88]. Additionally, 2-MTHF is employed as a solvent in polymer characterization methods [89,90] and plays a role in polymer recycling processes by aiding in the dissolution and separation of specific polymers from mixed plastic waste for material recovery [91,92]. In extraction and separation processes, 2-MTHF is utilized for its solvating properties, finding application in the extraction of natural products and the purification of organic compounds. It serves as a chromatographic solvent in both high-performance liquid chromatography (HPLC) and gas chromatography (GC), facilitating the separation of diverse compounds [93]. Additionally, 2-MTHF acts as an extraction solvent for isolating natural products, pharmaceutical compounds, and other target molecules from complex matrices [94], and is employed in liquid-liquid extraction processes to separate components based on their differential solubility in the organic phase [78,95].

## 3. Catalytic Conversion of LA to 2-MTHF

In this segment, an in-depth analysis is presented on the catalytic systems reported for the hydro-conversion of LA to 2-MTHF. The discussion encompasses diverse catalytic systems, explores the impact of parameters and co-catalysts, and delves into the intricacies of the reaction mechanism.

Upare et al. reported vapor phase hydrocyclization of LA to 2-MTHF using Nanocomposite Copper/Silica Catalyst in the presence of 1,4-dioxane at 265 °C and moderate H_2_ pressure [53]. In this study, the impact of H_2_ pressure and the weight percentage of the metal catalyst on 2-MTHF selectivity was investigated. At 265 °C and 1 bar H_2_ pressure, the 5 wt% Cu/SiO_2_ catalyst achieved full LA conversion, with 94% selectivity towards Gvl. Elevating the H_2_ pressure to 10 bar resulted in a substantial increase in Gvl selectivity, reaching 99.9%. However, with further pressure increments, the selectivity shifted towards 1,4-PDO by reducing the Gvl selectivity to 93%. To attain the selectivity of 2-MTHF, the authors studied wt% of Cu on SiO_2_ and found that increasing the wt% of Cu (from 5 wt% to 80 wt%) increased the selectivity of 2-MTHF and observed 64% of 2-MTHF at 80 wt% Cu/SiO_2_ (Table 1, entry 1 to 4). The highest selectivity of 2-MTHF (89%) resulted from the addition of Ni-metal to the Cu/SiO_2_ (8 wt% Ni–72 wt% Cu/SiO_2_), here the Ni metal act as a promoter and which enhanced the selectivity of 2-MTHF (Table 1, entry 5). Surprisingly, the Ni-Cu(72)/SiO_2_ exhibited exceptional selectivity towards 2-MTHF, reaching 89% without any notable decline in catalytic activity over a period of 320 h. This result highlights the advantageous impact of nickel on enhancing selectivity. Moreover, the leaching of Cu metal is not observed in this reaction and exhibits minimal sintering of metals during the reaction, which was observed in various physicochemical techniques.

The Luque research group successfully developed a method for producing 2-MTHF from LA using both microwave and continuous flow processes, eliminating the need for molecular hydrogen. In this innovative approach, formic acid was employed as a hydrogen source [96]. Notably, formic acid was utilized as a co-product generated during the preparation of LA from glucose. Formic acid is an alternative source to produce in-situ H_2_ by its decomposition during the reaction. Under microwave irradiation (150 °C temperature, 30 min microwave irradiation, 300 W), the authors screened several catalysts for this conversion, such as Ru-Starbon^®^, Rh-Starbon^®^, Pd-Starbon^®^, Cu-MINT, and Pd/C, among the catalysts used, the Cu-MINT catalyst showed high conversion, and Pd-based catalysts showed high selectivity towards 2-MTHF (Table 1, entry 6–10). Under continuous flow conditions, the Cu-MINT and Pd/C exhibited similar activities (79% and 73% conversion of LA) and showed significantly different selectivities toward 2-MTHF. In the Cu-MINT system, only two primary products were observed: 2-MTHF and 1,4-PDO, with selectivities of 60% and 40%, respectively. In contrast, the Pd/C system, though equally active, yielded a range of hydrogenation products, including 1,4-PDO, pentanoic acid (PA), and 4-hydroxyvaleric acid (HVA), with a limited selectivity towards 2-MTHF (<30 mol%). Further, they studied the stability of the Cu-MINT catalyst under a continuous process, and the catalyst experienced significant deactivation over time on stream, rendering it unstable in these flow conditions. Further studies on catalyst leaching revealed the presence of substantial amounts of leached copper in the solution (identified by ICP). Notably, these leached copper species exhibited a light blue color typical of Cu^2+^ ions, providing clear evidence of catalyst deactivation through leaching.

The Miller research group introduced homogenous catalysts (Ru-based complexes) for catalyzing the stepwise hydrogenation of levulinic acid (LA) to produce 2-methyltetrahydrofuran (2-MTHF) through intermediates Gvl and 1,4-PDO [97]. Two distinct catalytic systems employing branched triphosphine ligands, namely, Triphos (CH_3_C(CH_2_PPh_2_)_3_) and Ntriphos (N(CH_2_PPh_2_)_3_), were investigated. Among these, the most effective catalyst was found to be the pre-formed ruthenium species [RuH_2_(PPh_3_)-{N(CH_2_PPh_2_)_3_-κ_3_P}]. This catalyst accomplished almost total conversion of LA to 1,4-PDO without requiring any acidic additives, and it generated 87% 2-MTHF when employed in combination with HN(Tf)_2_ (Table 1, entry 11). Various acidic additives were examined to enhance the conversion of 1,4-PDO to 2-MTHF. Among them, HN(Tf)_2_ was the sole effective additive, while NH_4_PF_6_ and paratoluenesulfonic acid (p-TsOH) had adverse effects.

Obregûn et al. described the effectiveness of Ni- and Cu-based catalysts in converting LA to 2-MTHF in the presence of eco-friendly solvents such as H_2_O, ethanol, 1-butanol, and 2-propanol at 250 °C and 70 bar H_2_ pressure over 5 h (Table 1, entries 12 to 15) [52]. Among these solvents, 2-propanol stood out as the most efficient, owing to its favorable thermodynamic dehydrogenation pathway, which is crucial for providing adequate hydrogen for the reaction. Unlike the other alcohols and H_2_O medium, 2-propanol yielded the highest corresponding 2-MTHF. It has already been proven that Ni-based catalysts are known for their proficiency in dehydrogenating secondary alcohols into ketones and hydrogen, benefitted significantly from 2-propanol’s unique properties. From the experimental results, the authors revealed that, the Ni/Al_2_O_3_ represents the most potent catalyst for converting LA, while Cu/Al_2_O_3_ exhibited exceptional selectivity, achieving a 75% MTHF yield after 24 h of reaction time (Table 1, entries 15 and 16). The study also explored the synergetic effects of bimetallic Ni-Cu/Al_2_O_3_ catalysts, revealing a 56% MTHF yield in just 5 h at 250 °C under optimal Ni/Cu ratio conditions (Table 1, entry 17). These bimetallic catalysts demonstrated superior activity and selectivity, attributed to the formation of mixed Ni-Cu particles, which played a pivotal role in enhancing catalytic activity and ultimately improving the desired product’s yield.

**Scheme 5:**
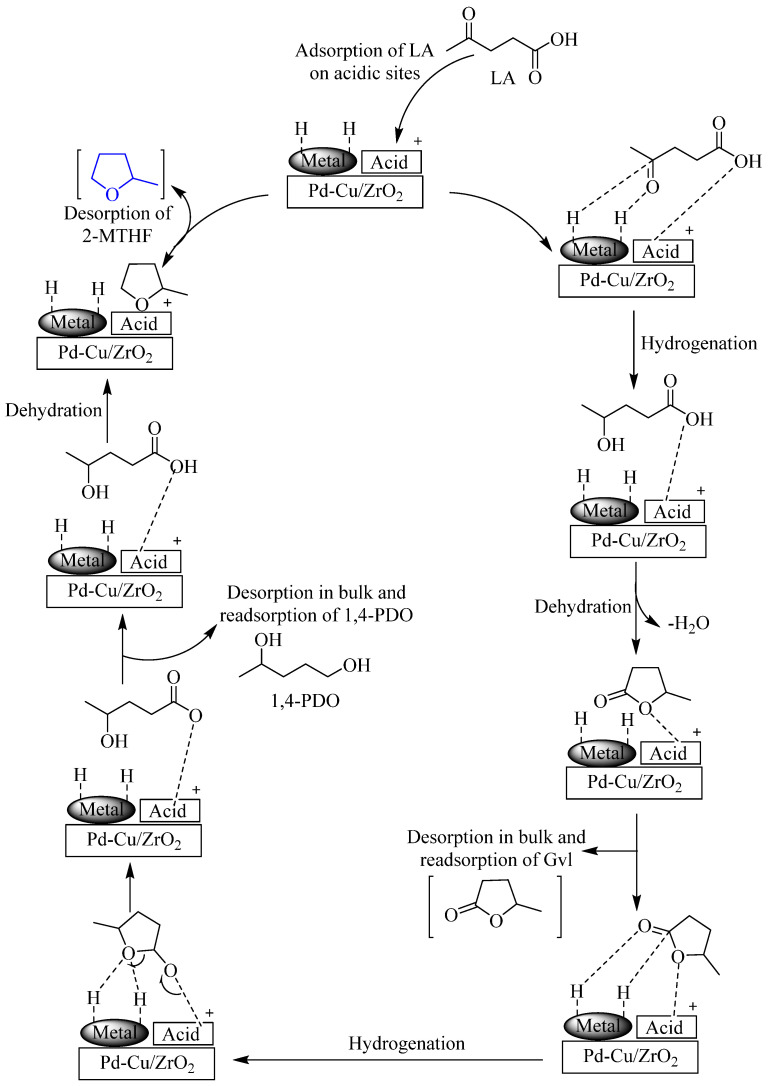
Synthesis Mechanism of Gvl, 1,4-PDO, and 2-MTHF from LA using a Pd-Cu/ZrO_2_ Catalyst. Scheme adopted from [98].

Patankar and Yadav showed the hydrocyclization of LA into Gvl, 1,4-PDO, and 2-MTHF utilizing a Pd-Cu/ZrO_2_ catalyst in an aqueous environment, employing relatively mild reaction conditions (200 °C, 60 bar H_2_). Nonetheless, the catalyst exhibited notably low selectivity for 2-MTHF (Table 1, entry 18) [98]. In the case of the mono-metal catalytic system (Cu/ZrO_2_) authors observed leaching. This leaching was effectively prevented by adding palladium to the Cu/ZrO_2_ catalyst, forming a stable copper-palladium alloy. The resulting Pd-Cu/ZrO_2_ catalyst demonstrated robustness and stability at high temperatures and pressures. The impact of different supports was investigated in this work using Pd-Cu/HT, Pd-Cu/Al_2_O_3_, and Pd-Cu/HMS catalysts (Table 1, entries 19 to 21). However, successful cascade synthesis to produce PDO and 2-MTHF from LA was accomplished using the Pd-Cu/ZrO_2_ catalyst due to its larger pore size and acidity, enabling the desired conversions. The authors clearly explained the role of metal sites and acidic sites of support in the effective formation of 2-MTHF (Scheme 5).

**Scheme 6:**
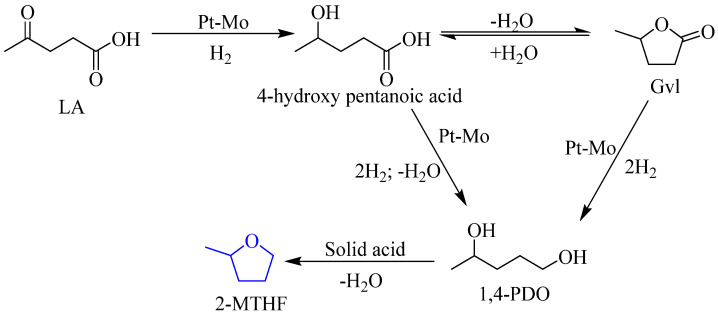
One-pot transformation of LA to 2-MTHF over Pt−Mo/H-β. Scheme adopted from [99].

Kaneda research group demonstrated the preparation of 2-MTHF from LA, efficiently facilitated in a one-pot process using a Pt-Mo bimetallic catalyst supported on H-β-zeolite, all in the presence of water and without any additional additives [99]. This catalyst system enables the hydrogenation of levulinic acid into 1,4-PDO, which is subsequently dehydrated to yield 2-THF by the H-β zeolite in an aqueous environment. The catalyst showed >99% conversion of LA with 86% of 2-MTHF under mild reaction condition i.e., 130 °C, 50 bar H_2_ (Table 1, entry 22). The reusability of catalysts is indeed crucial in the chemical industry, especially when working with precious metal catalysts. Precious metals, such as Pt, Pd, Rh, and others, are often used as catalysts in various industrial processes due to their high catalytic activity and selectivity. However, these metals are expensive, making it economically advantageous to recover and reuse them in multiple reaction cycles. In this work, the catalyst (Pt−Mo/H-β) was recovered and reused for the next two cycles. It showed remarkable activity and maintained the conversion of LA and selectivity of 2-MTHF, similar to the fresh catalyst (Table 1, entries 23 and 24). As per the experimental results, the authors declared that the catalyst support, such as H-β serves a dual function. First, it acts as a distinctive solid acid catalyst, facilitating the cyclodehydration of 1,4-pentanediol to 2-methyltetrahydrofuran in a water environment. Second, it functions as a support, enabling a synergistic interaction between Pt nanoparticles and MoOx species (Scheme 6). This synergy efficiently promotes the hydrogenation of levulinic acid to 1,4-pentanediol.

**Scheme 7:**
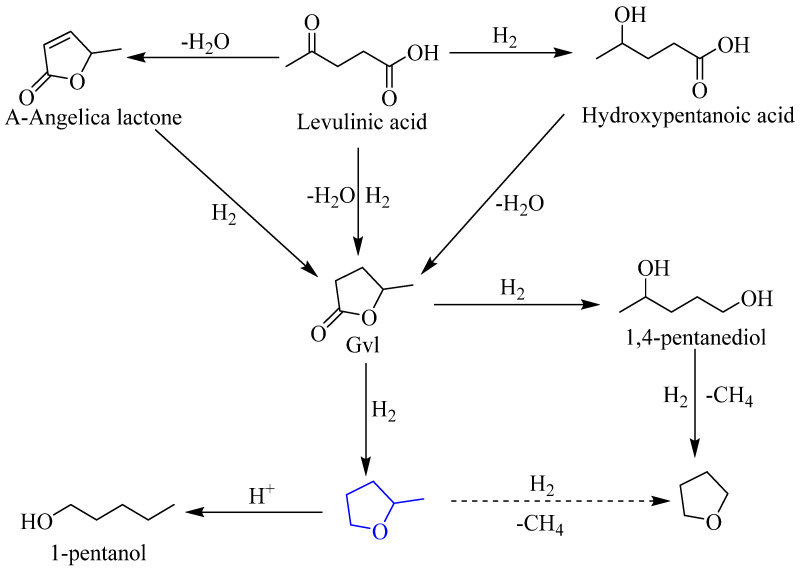
Reaction network of LA to tetrahydrofuranic compounds using Ru/GO catalyst. Scheme adopted from [100].

Upare et al. reported graphene oxide (GO) supported ruthenium nanoparticles (Ru/GO) for the hydrogenation of LA to produce cyclic ethers in a fixed-bed reactor (vapor-phase) [100]. Remarkably, Ru/GO catalysts exhibited a diverse range of hydrogenation products, including cyclic ethers (2-THF and tetrahydrofuran-THF at 54%) and Gvl at 41%. In order to increase the production of cyclic ethers, a two-step hydrogenation procedure was effectively employed to convert LA to Gvl. This method involved using Gvl as a secondary feedstock, enabling Ru/GO catalysts to generate cyclic ethers, including 2-MTHF and THF. The Ru/GO catalysts displayed exceptional selectivity, with an impressive 92% preference for cyclic ethers, notably achieving 77% selectivity for 2-MTHF, as evidenced in the two-step process (Table 1, entry 25). The outstanding activity and selectivity of LA hydrogenation over Ru/GO were attributed to the well-dispersed Ru nanoparticles and favorable interactions with GO, facilitated by the presence of oxy-functional groups (-carboxyl, -epoxy, -hydroxyl, etc.) on GO. Gvl was unequivocally established as the primary intermediate in 2-MTHF synthesis from LA, and there are two prominent routes for MTHF production from Gvl. The first step entails the hydrogenation of LA’s carbonyl group to produce Gvl on metallic catalyst sites. The second step involves the hydrogenation of Gvl to 1,4-PDO, also on metallic catalyst sites, followed by dehydration to form 2-MTHF with the assistance of acidic sites (Scheme 7). Under reduced hydrogen pressure, the major product obtained with Ru/GO catalysts was 1,4-PDO instead of 2-MTHF. Conversely, increasing the hydrogen pressure facilitated the formation of 2-MTHF, with 1,4-PDO acting as an intermediate (Table 1, entries 26 and 27).

**Scheme 8:**
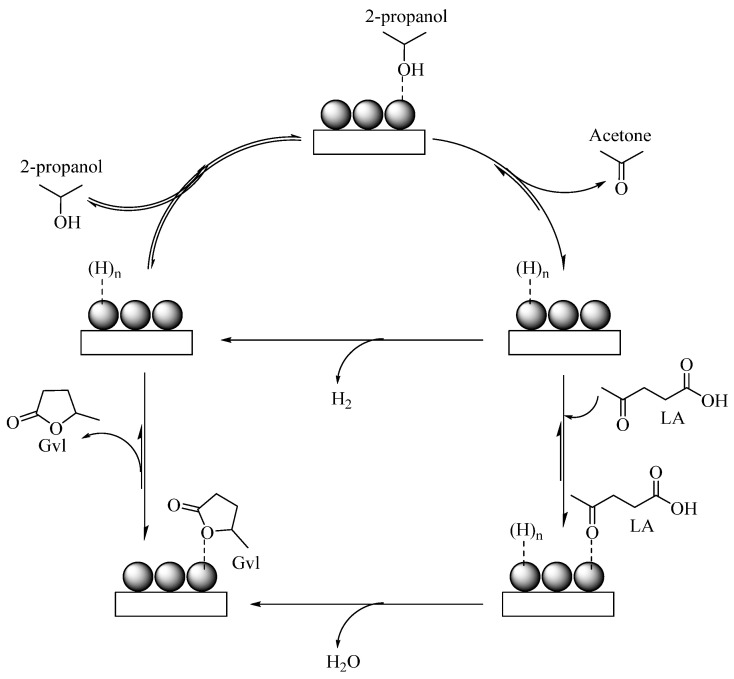
Catalytic transfer hydrogenation of LA in the presence of N_2_ atmosphere. Scheme adopted from [50].

**Scheme 9:**
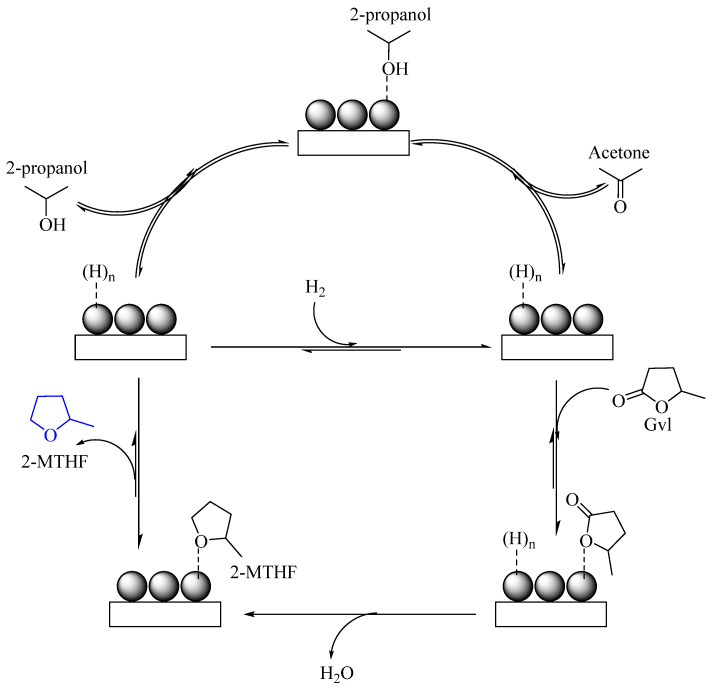
Catalytic transfer hydrogenation of LA in the presence of H_2_ atmosphere. Scheme adopted from [50].

Obregon et al. investigated two potential sources of hydrogen, molecular H_2_ (catalytic hydrogenation) and alcohols (catalytic transfer hydrogenation-CTH), for the transformation of LA to 2-MTHF over Ni-Cu/Al_2_O_3_ catalysts [50]. While individual hydrogenation through either CTH or molecular H_2_ alone yielded Gvl (80–90%) in amounts ranging, the production of MTHF was minimal (less than 3%) under moderate reaction conditions (5 h, 40 bar H_2_, 250 °C). However, under these reaction conditions, combining both hydrogen sources was essential to achieve a substantial yield of 2-MTHF (40%) (Table 1, entry 28). Indeed, it was observed that the presence of molecular H_2_ pressure had a beneficial effect on the reaction. This increased the concentration of dissolved hydrogen in the reaction medium, thereby decreasing hydrogen desorption from the catalyst surface. This increased availability of hydrogen greatly facilitated the efficient conversion of Gvl, resulting in a notable yield of MTHF. Additionally, the study established a direct correlation between the results in nitrogen (N_2_) and hydrogen (H_2_) atmospheres, underscoring the crucial role of catalytic hydrogenation in the conversion of LA to MTHF, even under high H_2_ pressure (Scheme 8 and Scheme 9). In the N_2_ atmospheric reaction case, only a trace amount of 2-MTHF is observed, and in the H_2_ atmospheric case, a moderate yield of 2-MTHF was observed. The hydrogen pressure amplifies the amount of dissolved H_2_ in the reaction medium. This increase facilitates the adsorption of hydrogen atoms while reducing their desorption rate, which is not observed in the case of N_2_ atmosphere (Table 1, entry 28 and 29). In summary of this work, employing Ni-Cu/Al_2_O_3_ catalyst in conjunction with a proficient hydrogen donor solvent (2-propanol), and operating under an H_2_ atmosphere, yielded impressive 2-MTHF rates of up to 80% after a 20 h reaction period (Table 1, entry 30).

**Scheme 10:**
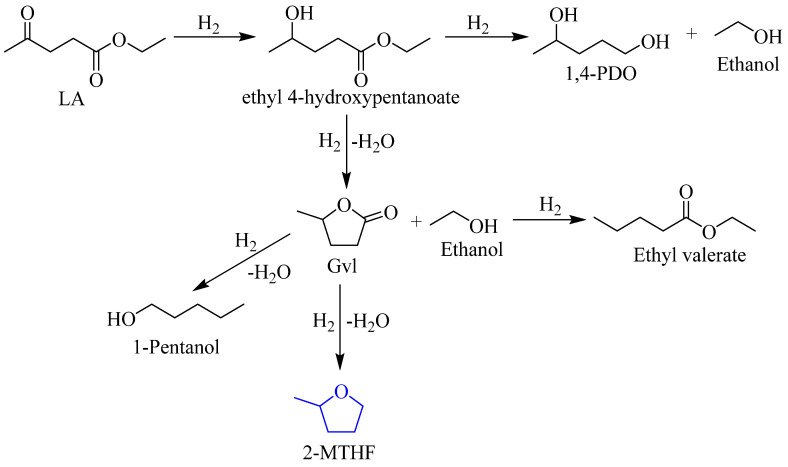
Hydro-conversion of Ethyl levulinate to 2-MTHF. Scheme adopted from [101].

Zheng et al. investigated the effect of reaction temperature on the hydro-conversion of ethyl levulinate (EL) under continuous reaction conditions using Cu/Al_2_O_3_-SiO_2_ catalyst [101]. At 140 °C, only 79% of EL was converted, with an impressive Gvl selectivity of 96%, and no quantity of 2-MTHF is observed at this temperature (Table 1, entry 31). Increasing the temperature to 150 °C enhanced EL conversion to 98% and remained the Gvl selectivity with a minimal 0.25% formation of 2-MTHF, and at 200 °C, 2-MTHF was observed with 9% selectivity (Table 1, entries 32 and 33). At lower reaction temperatures, effectively suppress the formation of undesired byproducts such as ring opened products (pentanol, pentanediol, ethyl pentanoate, etc.) (Scheme 10). However, with a stepwise increase in temperature, significant changes in selectivity were observed. The selectivity towards 2-MTHF increased at the expense of Gvl. At temperatures as high as 250 °C, 2-MTHF became the primary product, with 65% selectivity (Table 1, entry 34). At this temperature, the selectivity of Gvl is reduced to 8%, and the selectivity for 1-pentanol and ethyl valerate reached 17% and 4%, respectively.

**Scheme 11:**
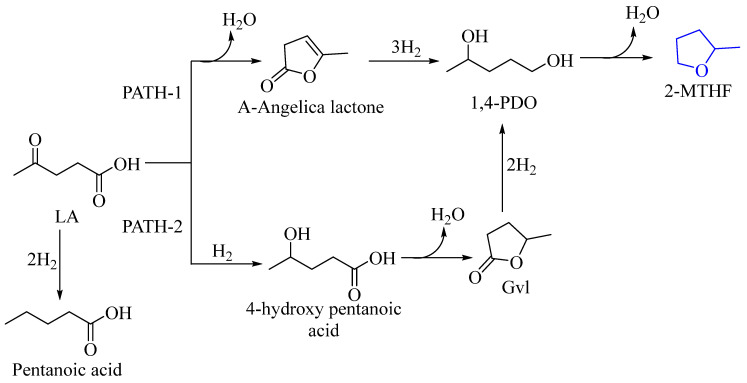
Conversion of LA to 2-MTHF using Ru/C + Re/C + NBP multi-catalytic system. Scheme adopted from [102].

**Scheme 12:**
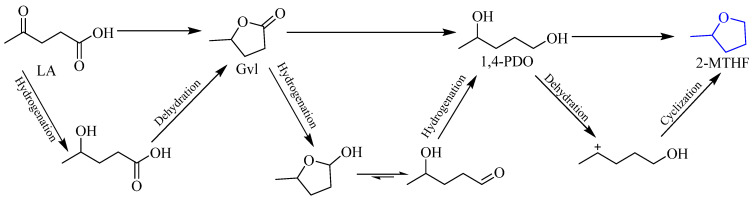
Stepwise reactions (hydrogenation and dehydration) during the conversion of LA to 2-MTHF using functional catalysts (Metal on acidic supports). Scheme adopted from [102].

Galletti and team explored the application of carbon-supported ruthenium (Ru) and rhenium (Re) metal catalysts in conjunction with niobium phosphate as an acid co-catalyst. This approach was devised to enhance selectivity in generating 2-MTHF from LA and Gvl [102]. In this research, the integration of a commercially available rhenium-based catalyst (10% Re/C) into an established catalytic system comprising Ru/C and NBP was examined. When employed independently, the Re/C catalyst showed no activity in the hydroconversion of Gvl. Nevertheless, in conjunction with [Ru/C + NBP], the Re/C catalyst demonstrated a synergistic effect, amplifying both Gvl conversion and selectivity towards 2-MTHF (Table 1, Entry 35). This synergy was observed even at low Re/C concentrations, highlighting the crucial role of rhenium in promoting the cyclization of diols to 2-MTHF in the reaction. The acidity of the co-catalyst (NBP) played a significant role in transforming LA and Gvl into 2-MTHF. The acidic nature of the co-catalyst facilitated interaction with Gvl’s carboxylic group, leading to its ring opening and subsequent conversion. Furthermore, the introduction of an acid co-catalyst (NBP) into [Ru/C + Re/C] systems was explored due to its known ability to activate not only lactone ring opening but also the internal dehydration of 1,4-PDO (intermediate in the reaction) to 2-MTHF (Scheme 11). The same catalyst was also employed for the hydroconversion of LA to 2-MTHF, resulting in 100% conversion of LA with 28% selectivity towards 2-MTHF (Table 1, Entry 36). The stepwise reactions, such as hydrogenation and dehydration occurring during the conversion of LA to 2-MTHF using functional catalysts (metal on acidic supports), are depicted in Scheme 12.

Mihályi research group studied solvent-free hydroconversion of LA using Co/SiO_2_ catalyst in a continuous process using a fixed-bed microreactor [103]. The catalyst is a bifunctional catalyst that consists of Co metal (for hydrogenation/hydrogenolysis reaction) and CoO_x_ Lewis acid active sites (dehydration reaction). During the reaction, the LA was initially dehydrated to create an angelica lactone (AL) intermediate. The selectivity of the reaction was influenced by the hydrogenation/hydrogenolysis activity of the catalyst, given the ease of LA dehydration. Under steady-state conditions at 200 °C and 30 bar pressure, the catalyst solely saturated the double bond of the AL ring, resulting in a 98 mol% yield of Gvl at complete LA conversion. However, at 225 °C, the catalyst’s high hydrogenation activity led to the cleavage of the Gvl ring, yielding 2-MTHF with a stable yield of approximately 70 mol% (Table 1, entry 37 and 38). These findings indicate that at 200 °C, the reaction produces a catalyst surface that is both active and enduring in selectively generating Gvl from LA. However, the delicate equilibrium between hydrogenation and dehydration activities, which accounts for the high Gvl selectivity, becomes precarious. With a slight increase in temperature, the heightened hydrogenation activity transforms Gvl into 2-MTHF. FT-IR spectroscopic analysis of adsorbed LA indicated the formation of H-bound LA and surface carboxylates. Surface intermediates, such as 4-hydroxy-3-pentenoate and 4-hydroxypentanoate, were identified during Gvl formation through dehydration.

Xie et al. screened several metal catalysts for this conversion under batch reaction conditions (220 °C, 30 bar H_2_, 10 h) in the presence of 2-butanol medium [104]. The Pd/C and Pt/C catalysts showed selectivity towards Gvl, the Ru/C gave good selectivity towards sec-butyl 4-oxopentanoate and in this case 2-MTHF was observed only 2%. The three above-mentioned catalysts have less selectivity for 2-MTHF. The mesoporous bifunctional and bimetallic such as Cu-Ni/Al_2_O_3_-ZrO_2_ (prepared from impregnation technique) exhibited excellent catalytic activity towards selective formation of 2-MTHF (92%) (Table 1, entries 39 to 42). The authors concluded that this exceptional catalytic performance by various physicochemical techniques can be attributed to the catalyst’s mesoporous structure, the acidic properties of the support, and the synergistic effects between Cu and Ni. Moreover, the Ni-Cu/Al_2_O_3_-ZrO_2_ catalyst demonstrated remarkable stability, retaining its catalytic activity and selectivity even after five reuse cycles. At optimal metal and support ratios, such as Cu (10 wt%)–Ni (10 wt%)/Al_2_O_3_-ZrO_2_ (9: Mole ratio of Al to Zr), which revealed complete conversion of LA to 2-MTHF (99.8%). In this work, we also studied the effect of reaction parameters on the reaction using Ni-Cu/Al_2_O_3_-ZrO_2_ catalyst, at below 180 °C, and the predominant product was Gvl. With a rise in temperature beyond this point, MTHF selectivity increased, while the Gvl yield notably dropped. Optimal MTHF selectivity (99.8%) was achieved at 220 °C. However, elevating the temperature further decreased MTHF selectivity due to the formation of by-products like sec-butyl valerate. At low hydrogen pressure, Gvl yield is favored, but increasing H_2_ pressure led to higher MTHF selectivity at the expense of Gvl selectivity. MTHF selectivity peaked at 99.8% when H_2_ pressure exceeded 30 bar H_2_ (Table 1, entry 43). A low reaction pressure hampered the ring-opening/cyclodehydration of Gvl into MTHF, resulting in decreased selectivity. Finally, the authors also studied the reusability activity of Ni/Al_2_O_3_-ZrO_2_, the catalyst displayed the ability to be reused up to five times with minimal loss in both catalytic activity and selectivity.

**Scheme 13:**
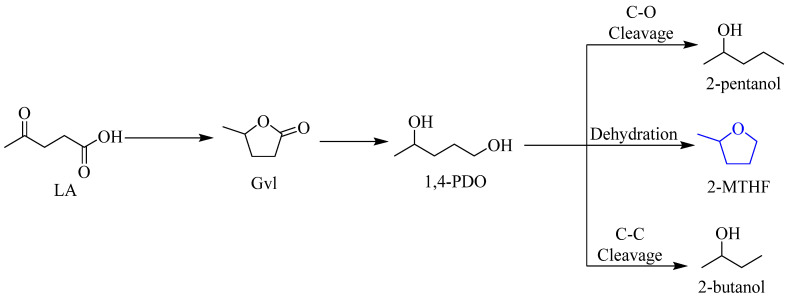
Reaction products of hydro-conversion of LA over NiCo/γ-Al_2_O_3_ catalyst. Scheme adopted from [105].

Gu et al. reported NiCo/γ-Al_2_O_3_ catalyst for the conversion of LA to 2-MTHF at various reaction parameters [105]. The proportions of Ni and Co in the NiCo/γ-Al_2_O_3_ catalyst were examined. Under optimal conditions, the Ni (5 wt%) Co (25 wt%)/γ-Al_2_O_3_ catalyst exhibited significant activity, achieving a 73% yield of 2-MTHF (Table 1, entry 44). X-ray Photoelectron Spectroscopy (XPS) analysis revealed a higher presence of metal oxides on bimetallic catalysts, contrasting with reduced metals (M(0)) predominantly found on monometallic counterparts. Additionally, bimetallic catalysts exhibited heightened acidity compared to their monometallic counterparts, and the presence of metal oxide content in bimetallic catalysts could be the reason for the high acidity of the bimetallic catalyst. Actually, the acidity of the catalyst plays an important role in the conversion of LA to 2-MTHF; the appropriate acidic sites/strength in bimetallic Ni-Co/γ-Al_2_O_3_ is the main reason for the highest yield of 2-MTHF as per monometallic catalysts (Table 1, entries 44 to 46). The maximum yield of Gvl reached 95% at 190 °C and exhibited a gradual decline with increasing temperatures. Conversely, the yield of 2-MTHF rose from 0.3% at 190 °C to 73% at 240 °C. A slight decrease to 71% in 2-MTHF yield occurred when the temperature was further increased to 250 °C, indicating that elevated temperatures might induce undesired side reactions. At a H_2_ pressure of 10 bar, the yield of 2-MTHF was 33%, with Gvl being the primary product at 54%. As the H_2_ pressure increased from 10 bar H_2_ to 50 bar H_2_, there was a gradual rise in the yield of 2-MTHF, reaching 73% at 50 bar H_2_. However, further increasing the pressure to 60 bar H_2_ did not enhance the 2-MTHF yield, indicating a saturation point for the surface M-H (M = Ni and Co) species at 50 bar H_2_. Additionally, there was a gradual increase in the yield of 2-butanol (C-C cleavage of 1,4-PDO intermediate), underscoring the competitive nature between the production of 2-MTHF and monoalcohol (Scheme 13). In the initial hour, a low yield of 2-MTHF at 12% was observed, with Gvl being the primary product at 76%. This indicated an insufficient reaction time for the production of 2-MTHF. With an extended reaction time of 5 h, the 2-MTHF yield increased to 73%, and there was also a rise in the yield of 2-butanol. Further extending the reaction time to 6 h resulted in a slight decrease in 2-MTHF yield (72%), while the yield of 2-butanol increased, which means after 6 h the conversion initiates C-C cleavage to form alcohol products. The authors also investigated reuse of the NiCo/γ-Al_2_O_3_ catalyst, four consecutive cycles of LA to 2-MTHF were conducted at 240 °C, 5 bar H_2_ for 4 h; there was a slight decrease in the conversion of LA and the yield of 2-MTHF. The comparative yield of 2-MTHF decreased from 66% to 63% over these four runs, with the conversion of LA gradually decreasing from 92% to 87%. This reusability experiment suggests that the NiCo/γ-Al_2_O_3_ exhibits promising stability and reusability.

The Seames research group demonstrated the conversion of LA to 2-MTHF in two steps: 1. Catalytic Transfer Hydrogenation (isopropanol as a hydrogen source) of LA to Gvl using Zr containing β-zeolite catalyst, 2. Catalytic hydro-conversion of Gvl (obtained in the first step) to using CuO/Al_2_O_3_ catalyst in the presence of a molecular H_2_ source at batch and continuous operations [46].

In the first step the Zr-β-zeolite catalyst showed 97% selectivity towards the Gvl at relatively milder reaction conditions (Table 1, entry 47).

The optimal conditions for maximizing the conversion of Gvl to 2-MTHF in the second reaction were established as follows:

- In batch reaction conditions, the conversion of Gvl to 2-MTHF shows an 84% yield (96% of sel.) using CuO/Al_2_O_3_ catalyst (Table 1, entry 48).
- In fixed-bed reaction conditions (continuous process), the conversion of Gvl to 2-MTHF shows a 93% yield (98% of sel.) using CuO/Al_2_O_3_ catalyst (Table 1, entry 49).

The reaction temperature 200 °C showed maximum selectivity towards 2-MTHF; at lower temperatures, the reaction favored the formation of 1,4-PDO, but this effect could be mitigated by extending the residence time. At reaction temperatures of 250 °C or higher led to unwanted side reactions such as ring opening products.

**Table 1:** Catalytic conversion of LA to 2-MTHF.

S. No	Catalyst	Reactant	Reaction Conditions (Temp., H_2_ Pressure, Time in Hours)	Conv. (%)	2-MTHF sel. (%)	Ref.
1	5 wt% Cu/Al_2_O_3_	LA	265 °C, H_2_ (10 bar), time (5 h), *p*-dioxane, fixed-bed reactor (vapour phase)	100	0.1	[53]
2	30 wt% Cu/Al_2_O_3_	LA	265 °C, H_2_ (10 bar), time (5 h), *p*-dioxane, fixed-bed reactor (vapour phase)	100	3	[53]
3	50 wt% Cu/Al_2_O_3_	LA	265 °C, H_2_ (10 bar), time (5 h), *p*-dioxane, fixed-bed reactor (vapour phase)	100	43	[53]
4	80 wt% Cu/Al_2_O_3_	LA	265 °C, H_2_ (10 bar), time (5 h), *p*-dioxane, fixed-bed reactor (vapour phase)	100	64	[53]
5	8 wt% Ni-72 wt% Cu/SiO_2_	LA	265 °C, H_2_ (10 bar), time (5 h), *p*-dioxane, fixed-bed reactor (vapour phase)	100	89	[53]
6	Ru-Starbon^®^	LA	150 °C, time (30 min), 300 W (microwave irradiation), FA as H_2_ source	30	50	[96]
7	Rh-Starbon^®^	LA	150 °C, time (30 min), 300 W (microwave irradiation), FA as H_2_ source	69	90	[96]
8	Pd-Starbon^®^	LA	150 °C, time (30 min), 300 W (microwave irradiation), FA as H_2_ source	64	88	[96]
9	Cu-MINT	LA	150 °C, time (30 min), 300 W (microwave irradiation), FA as H_2_ source	90	75	[96]
10	Pd/C	LA	150 °C, time (30 min), 300 W (microwave irradiation), FA as H_2_ source	78	92	[96]
11	Ru[H_2_CON(PPh_2_)_3_]	LA	150 °C, H_2_ (65 bar), 25 h, THF, batch reactor	100	87	[97]
12	Ni/Al_2_O_3_	LA	250 °C, H_2_ (70 bar), 5 h, H_2_O, batch reactor	100	1.4	[52]
13	Ni/Al_2_O_3_	LA	250 °C, H_2_ (70 bar), 5 h, ethanol, batch reactor	100	0.5	[52]
14	Ni/Al_2_O_3_	LA	250 °C, H_2_ (70 bar), 5 h, 1-butanol, batch reactor	93	10	[52]
15	Ni/Al_2_O_3_	LA	250 °C, H_2_ (70 bar), 5 h, 2-propanol, batch reactor	100	46	[52]
16	Cu/Al_2_O_3_	LA	250 °C, H_2_ (70 bar), 24 h, 2-propanol, batch reactor	100	75	[52]
17	Ni-Cu/Al_2_O_3_	LA	250 °C, H_2_ (70 bar), 5 h, 2-propanol, batch reactor	100	56	[52]
18	Pd-Cu/ZrO_2_	LA	200 °C, 50 bar H_2_, 24 h, H_2_O, batch reactor	100	25% 1,4-PDO 5% 2-MTHF	[98]
19	Pd-Cu/HT	LA	200 °C, 50 bar H_2_, 24 h, H_2_O, batch reactor	100	Not observed	[98]
20	Pd-Cu/Al_2_O_3_	LA	200 °C, 50 bar H_2_, 24 h, H_2_O, batch reactor	100	Not observed	[98]
21	Pd-Cu/HMS	LA	200 °C, 50 bar H_2_, 24 h, H_2_O, batch reactor	100	Not observed	[98]
22	Pt−Mo/H-β	LA	150 °C, 50 bar H_2_, 12 h, H_2_O, batch reactor	>99	86	[99]
23	Pt−Mo/H-β (reused—1st Cycle)	LA	150 °C, 50 bar H_2_, 12 h, H_2_O, batch reactor	>99	85	[99]
24	Pt−Mo/H-β ((reused—2nd Cycle)	LA	150 °C, 50 bar H_2_, 12 h, H_2_O, batch reactor	>99	85	[99]
25	5% Ru/GO	Gvl (obtained from LA)	265 °C, 25 bar H_2_, 1,4-dioxane in fixed-bed reactor	100	92% (77% 2-MTHF and 15% THF)	[100]
26	5% Ru/GO	Gvl (obtained from LA)	265 °C, 10 bar H_2_, 1,4-dioxane in fixed-bed reactor	69	83% PDO	[100]
27	5% Ru/GO	Gvl (obtained from LA)	265 °C, 1 bar H_2_, 1,4-dioxane in fixed-bed reactor	48	92% PDO	[100]
28	Ni-Cu/Al_2_O_3_	LA	250 °C, 40 bar H_2_, 5 h, 2-Propanol, batch reactor	100	40	[50]
29	Ni-Cu/Al_2_O_3_	LA	250 °C, 40 bar N_2_, 5 h, 2-Propanol, batch reactor	100	<5	[50]
30	Ni-Cu/Al_2_O_3_	LA	250 °C, 40 bar H_2_, 20 h, 2-Propanol, batch reactor	100	80	[50]
31	Cu/Al_2_O_3_-SiO_2_	EL	140 °C, 15 bar H_2_, ethanol, fixed-bed reactor	79	Nil	[101]
32	Cu/Al_2_O_3_-SiO_2_	EL	150 °C, 15 bar H_2_, ethanol, fixed-bed reactor	98	0.25	[101]
33	Cu/Al_2_O_3_-SiO_2_	EL	200 °C, 15 bar H_2_, ethanol, fixed-bed reactor	99	9	[101]
34	Cu/Al_2_O_3_-SiO_2_	EL	250 °C, 15 bar H_2_, ethanol, fixed-bed reactor	100	65	[101]
35	Ru/C + Re/C + NBP	Gvl	200 °C, 90 bar H_2_, 3 h, H_2_O, batch reactor	40	65	[102]
36	Ru/C + Re/C + NBP	LA	180 C, 50 bar H_2_, 3 h, H_2_O, batch reactor	100	28	[102]
37	Co/SiO_2_	LA	200 °C, 30 bar H_2_, No solvent, fixed-bed reactor	100	trace	[103]
38	Co/SiO_2_	LA	225 °C, 30 bar H_2_, No solvent, fixed-bed reactor	100	70	[103]
39	5% Pd/C	LA	220 °C, 30 bar H_2_, 10 h, 2-butanol, batch reactor	100	6.4	[104]
40	5% Pt/C	LA	220 °C, 30 bar H_2_, 10 h, 2-butanol, batch reactor	100	10	[104]
41	5% Ru/C	LA	220 °C, 30 bar H_2_, 10 h, 2-butanol, batch reactor	100	2.6	[104]
42	Cu (5%)-Ni (10%)/Al_2_O_3_-ZrO_2_ (9)	LA	220 °C, 30 bar H_2_, 10 h, 2-butanol, batch reactor	100	92.3	[104]
43	Cu (10%)-Ni (10%)/Al_2_O_3_-ZrO_2_ (9)	LA	220 °C, 30 bar H_2_, 10 h, 2-butanol, batch reactor	100	99.8	[104]
44	Ni-Co/γ-Al_2_O_3_	LA	250 °C, H_2_ (50 bar), 5 h, isopropanol, batch reactor	99.9	73	[105]
45	Co/γ-Al_2_O_3_	LA	250 °C, H_2_ (50 bar), 5 h, isopropanol, batch reactor	99.9	62	[105]
46	Ni/γ-Al_2_O_3_	LA	250 °C, H_2_ (50 bar), 5 h, isopropanol, batch reactor	99.9	17	[105]
47	Zr-β-zeolite	LA	170 °C, H_2_ (50 bar), 4 h, isopropanol, batch reactor	-	97 (88% yield) *	[46]
48	CuO/Al_2_O_3_	Gvl (obtained in 1st step)	200 °C, H_2_ (15 bar), 2 h, batch reactor	-	96 (84% yield)	[46]
49	CuO/Al_2_O_3_	Gvl (obtained in 1st step)	200 °C, time (residence) of 2.5 h, and a feed rate 10 mol% excess H_2_, 2-propanol, fixed-bed reactor	-	98 (93% yield)	[46]

* Yield and selectivity of Gvl.

## 4. Conclusions and Outlook

Synthesizing 2-methyltetrahydrofuran (2-MTHF) from levulinic acid (LA) presents a major challenge in bio-refinery processes. The significance of 2-MTHF in biofuel and its safe solvent applications in diverse industries has led to extensive research efforts to address this challenge. In batch reactions, the Pt−Mo/H-β catalyst demonstrated high efficiency, yielding 2-MTHF at an 85–86% rate from LA in an aqueous medium at 150 °C, the H-β support acidity in promoting synergistic interactions between Pt nanoparticles and MoO_x_ species were key findings. Another, i.e., Cu-Ni/Al_2_O_3_-ZrO_2_, with equivalent Cu-10 wt% and Ni-10 wt% on ZrO_2_ support, achieved the highest catalytic activity, yielding 99% of 2-MTHF in the presence of 2-butanol. Its exceptional performance was attributed to the acidic characteristics of the support and the synergistic interactions between Cu and Ni. The CuO/Al_2_O_3_ catalyst precursor yielded 85% 2-MTHF from Gvl at 200 °C, 15 bar H_2_ pressure, and 2 h. Similarly, the Ni-Cu/Al_2_O_3_ catalyst achieved an 80% yield of 2-MTHF from LA using molecular hydrogen and 2-butanol as hydrogen sources, with both sources enhancing selectivity towards 2-MTHF. The Ni-Co/γ-Al_2_O_3_ catalyst proved to be a highly effective bimetallic catalyst, achieving a 73% yield of 2-MTHF from LA and emphasizing the significance of suitable acidic sites. Under microwave irradiation, Rh-Starbon^®^, Pd-Starbon^®^, Cu-MINT, and Pd/C catalysts yielded 2-MTHF with selectivity ranging from 75% to 95% and moderate LA conversion.

In fixed-bed conditions, a high metal content catalytic system (8 wt% Ni and 72 wt% Cu on SiO_2_) achieved an excellent yield (89%) of 2-MTHF at 265 °C and 25 bar H_2_ pressure in a 1,4-dioxane medium, marking the first report of such a high yield under these conditions. Similarly, the copper-based catalyst Cu/Al_2_O_3_-SiO_2_ showed a substantial 65% yield of 2-MTHF from ethyl levulinate in an ethanol medium under comparable reaction conditions. A non-noble metal catalyst, Co/SiO_2_, demonstrated complete LA conversion with a remarkable 70% selectivity towards 2-MTHF under solvent-free conditions. Additionally, the Ru/GO catalyst exhibited a high yield of tetrahydrofurans (77% 2-MTHF and 15% THF) from LA at 265 °C and 25 bar H_2_ pressure in the presence of a 1,4-dioxane medium. This success was attributed to well-dispersed Ru nanoparticles and their favorable interactions with the graphene oxide (GO) support, facilitated by the presence of oxygen-functional groups, enhancing the catalytic activity of Ru/GO.

Several noteworthy aspects are involved in the transformation of LA into 2-MTHF. The introduction of co-metal to the catalyst enhances the selectivity of 2-MTHF, as exemplified by the utilization of Ni metal in the vapor phase with Cu/SiO_2_. The remarkable activity and selectivity demonstrated by catalysts in producing 2-MTHF can be credited to well-dispersed metal nanoparticles and favorable interactions facilitated by oxygen-functional groups (-carboxyl, -epoxy, -hydroxyl, etc.) on the support. This phenomenon is evident in the case of Ru/GO. An alcohol-based hydrogen source, such as 2-propanol, emerged as the most efficient solvent in the reaction, generating H_2_ through decomposition. The hydrogen produced was then employed in the conversion of LA to 2-MTHF. The Ni-Cu-supported catalyst exhibited remarkable catalytic activity for this conversion in the presence of 2-propanol. Temperature plays a pivotal role in LA conversion, with the range of 170–240 °C being conducive to 2-MTHF formation, while elevated temperatures result in the generation of ring-opening products. The pressure of hydrogen is a critical factor, as lower pressures promote Gvl yield, whereas higher pressures boost 2-MTHF selectivity. Bimetallic catalysts, characterized by heightened acidity attributed to metal oxides, exert a significant influence on the conversion of LA to 2-MTHF.

The conversion process faces challenges because of the use of hazardous solvents, excessive metal content in catalysts, limited selectivity of some catalysts for 2-MTHF, instability of catalytic materials, and low productivity of 2-MTHF. To overcome these problems, there is a need for a catalytic system that works in safer conditions and provides higher yields and throughput of 2-MTHF, all under relatively moderate reaction conditions. Tackling these challenges is a crucial and ambitious goal in bio-refinery research.

## Data Availability

Not applicable.
